# Snakes with Coordinate Regeneration Technique: An Application to Retinal Disc Boundary Detection

**DOI:** 10.1155/2013/852613

**Published:** 2013-10-05

**Authors:** Asloob Ahmad Mudassar, Saira Butt

**Affiliations:** ^1^Department of Physics and Applied Mathematics, Pakistan Institute of Engineering and Applied Sciences, P.O. Nilore, Islamabad 45650, Pakistan; ^2^Isotope Application Division, Pakistan Institute of Nuclear Science and Technology, P.O. Nilore, Islamabad 45650, Pakistan

## Abstract

A modified snake method based on the novel idea of coordinate regeneration is presented and is tested on an object with complex concavities and on retinal images for locating the boundaries of optic discs, where the conventional snake methods fail. We have demonstrated that the use of conventional snake method with our proposed coordinate regeneration technique gives ultimate solution for finding the boundaries of complex objects. The proposed method requires a Gaussian blur of the object with a large kernel so that the snake can be initialised away from the object boundaries. In the second and third steps the blurring kernel size is reduced so that exact boundaries can be located. Coordinate regeneration is applied at each step which ultimately converges the snake (active contour) to exact boundaries. For complex objects like optic discs in retinal images, vessels act as snake distracters and some preimage processing is required before the proposed technique is applied. We are demonstrating this technique to find the boundary of optic discs in retinal images. In principle, this technique can be extended to find the boundary of any object in other modalities of medical imaging. Simulation results are presented to support the idea.

## 1. Introduction

In this section, we are introducing a modified form of snake method used for automatic tracking of boundary of an object in a medical image. We are considering retinal images as medical images and optic disc as an object of interest. The first snake model was introduced by Kass et al. [1]. Mendels et al. [2] were the persons who applied this technique for the first time to track the boundary of the optic disc in retinal images. The concept of gradient vector flow (GVF) snake was introduced to extend the range of initialisation of snakes [3] to make the snake method more effective referred to as GVF snakes. We have made some changes to the normal snake method by introducing a new concept of coordinate regeneration on the curve and made it more effective. The main advantage of coordinate regeneration is to extend the range for initialisation of snakes around or within a boundary and to come out with a more precise boundary data. The algorithm based on coordinate regeneration is much faster and more robust when combined with normal snake method. Later in this paper, we will establish that the concept of coordinate regeneration alleviates the need of GVF snakes which has been a hot topic for more than a decade. For completeness, we are giving a brief introduction to the theory of normal and GVF-snake models, and thereafter, we shall present the novel ideal of coordinate regeneration. 

Snakes or active contours are parametric curves defined within an image domain that can move under the influence of internal forces arising from within the curve itself and external forces computed from the image data. These internal and external forces are defined in such a manner that the snake contour moves towards an object boundary. Snakes are extensively used in many applications such as edge detection [1], shape modelling [4, 5], segmentation [6, 7], motion tracking, and traffic monitoring [8]. 

Active contour models are classified as parametric [1] or nonparametric (geometric) [9–11] active contours. The parametric active contour is a contour that is represented by a small number of parameters that capture the shape of the object, whereas the geometric active contour has the ability to change curve topology during deformation. Parameterisation is achieved by expressing the curve as a weighted sum of a set of known functions. The parameter space is different from the physical space as the curve is initially defined in physical space and evolves over time. Different bases give parameter space different properties, and some operations that are performed on the contour can be defined in the parameter space. Parametric active contours synthesize parametric curves within an image domain and allow them to move towards desired features especially edges. The curves are driven towards the edges due to potential forces. Potential forces are defined as forces that can be expressed as a negative gradient of a potential function. These forces are extracted from the image data, whereas internal forces arise from the curve due to bending and elasticity. 

A traditional snake or active contour is a curve *X*(*s*) = [*x*(*s*), *y*(*s*)],  *s* ∈ [0,1] that moves through the spatial domain of an image in order to minimize the energy function given by:

(1) $$\begin{array}{c} E=\overset{1}{\underset{0}{\int }}\frac{1}{2}\left[\alpha {\left|X^{\prime }\left(s\right)\right|}^{2}+\beta {\left|X^{\prime \prime }\left(s\right)\right|}^{2}\right]+E_{\text{ext}}\left(X\left(s\right)\right)ds. \end{array}$$

In (1), parameters *α* and *β* control the tension and rigidity of the snake, respectively *X*′(*s*) and *X*′′(*s*) are the first and second order derivatives of *X*(*s*) with respect to *s*, and *E*_ext_ represents the external energy of the image, which can be computed from the image data. 

Let us consider a grey level image *I*(*x*, *y*) which is a function of position variables (*x*, *y*). Its external energy is expressed in the following ways [1]:

(2) $$\begin{array}{c} E_{\text{ext}}^{1}\left(x,y\right)=-{\left|\nabla I\left(x,y\right)\right|}^{2}, \end{array}$$

(3) $$\begin{array}{c} E_{\text{ext}}^{2}\left(x,y\right)=-{\left|\nabla \left\{G_{\sigma }\left(x,y\right)⊗I\left(x,y\right)\right\}\right|}^{2}. \end{array}$$

Here, ∇ is the gradient operator, and *G*_*σ*_(*x*, *y*) is a two-dimensional Gaussian function with standard deviation *σ*. If image is a line drawing (black on white), then external energies can be expressed as follows [12]:

(4) $$\begin{array}{c} E_{\text{ext}}^{3}\left(x,y\right)=I\left(x,y\right), \end{array}$$

(5) $$\begin{array}{c} E_{\text{ext}}^{4}\left(x,y\right)=G_{\sigma }\left(x,y\right)⊗I\left(x,y\right). \end{array}$$

From (3) and (5), it is easy to conclude that large values of *σ* will make the boundaries blur, but at the same time large *σ* are essential to increase the capture range of the snake. In short, *σ* is a trade-off parameter between the exact boundary localisation and the capture range. A snake that minimizes energy *E* must satisfy the Euler equation [1, 12]. 

Consider:

(6) $$\begin{array}{c} \alpha X^{\prime \prime }\left(s\right)-\beta X^{\prime \prime \prime \prime }\left(s\right)-\nabla E_{\text{ext}}=0. \end{array}$$

The above equation can be expressed as a force balance equation and can be written as

(7) $$\begin{array}{c} F_{\mathrm{int}}+F_{\text{ext}}^{p}=0, \end{array}$$

where *F*_int_ = *αX*′′(*s*) − *βX*′′′′(*s*) and *F*_ext_^*p*^ = −∇*E*_ext_. The internal force *F*_int_ opposes stretching and bending, and the external force *F*_ext_^*p*^ pulls the snake towards the desired edges. In order to solve (6), the snake is made dynamic by treating *X* as a function of time *t* as well as *s*, that is, *X*(*s*, *t*). Then, the partial derivative of *X* with respect to *t* is set equal to the left-hand side of (6). 

Consider:

(8) $$\begin{array}{c} X_{t}\left(s,t\right)=\frac{dX\left(s,t\right)}{dt}=\alpha X^{\prime \prime }\left(s\right)-\beta X^{\prime \prime \prime \prime }\left(s\right)-\nabla E_{\text{ext}}. \end{array}$$

When statistical equilibrium for *x*(*s*, *t*) is reached, then the term *X*_*t*_(*s*, *t*) will vanish, and the solution of (6) is obtained. 

There are two main difficulties associated with the traditional snake model. The first problem is its sensitivity to initialisation, as the snake needs to be initialised very close to the true boundaries; otherwise, it will most likely converge to the wrong results. In order to address the issue of wrong convergence, several methods have been proposed in the literature such as pressure forces [12] and distance potentials [13]. The main idea behind these forces is to increase the capture range of the external force field (as it is very strong near the edges and vanishes quickly in the homogeneous part of the image) to move the contour towards the desired boundary. 

The second problem associated with the traditional snake method is its inability to progress into boundary concavities [14]. Although pressure forces [12], control points [15], and the use of solenoidal fields [3] have been proposed, these methods solve only one aspect of the problem creating new difficulties. For example, pressure forces can push a snake into boundary concavities but cannot do it very strongly. Pressure forces must also be initialised to push in or push out which requires careful initialisation. 

The solution to all these problems faced by the use of traditional active contours or snakes was proposed by introducing a new kind of external force which addresses both the issues of short range external field and sensitivity to initialisation. These fields are called gradient vector flow (GVF) fields [16]. These are very strong fields derived from the images by an energy minimization process. This minimization is achieved by solving a pair of decoupled linear partial differential equations. These equations diffuse the gradient vectors of the grey level or binary edge maps computed from the image. An active contour or snake that uses the GVF field as the external force is called a GVF snake. Unlike pressure forces, GVF snakes do not need prior knowledge about whether to shrink or to expand. They have a long capture range, so they can be initialised far away from the boundaries. The large capture range is achieved through a computational diffusion process that does not affect the edges or concavities. The method of distance potential of Cohen is very similar to the GVF method, but this model is unable to move the snake into concavities. GVF snake is now considered to be the most efficient method. The subject will remain incomplete without describing the details related to GVF. The detail about GVF is presented in the next section. 

## 2. Gradient Vector Flow (GVF)

The poor convergence of the traditional snake is due to convergence of the solution to local minima. The solution to this problem as presented by [16] is to replace the standard external force *F*_ext_^*p*^ in (7) with a static external force that does not change with time or depend on the starting position of the snake. This new static external force field *F*_ext_^*g*^ = *V*(*x*, *y*) is called the gradient vector flow field or GVF field. Then, the external potential force in (8) is replaced with *V* and gives the following equation:

(9) $$\begin{array}{c} X_{t}\left(s,t\right)=\alpha X^{\prime \prime }\left(s\right)-\beta X^{\prime \prime \prime \prime }\left(s\right)+V. \end{array}$$

The parametric curve obtained by solving the above equation is known as the GVF snake. The GVF field points towards the object boundaries when very close to the boundary and varies smoothly over the homogeneous region of the image. The main advantage of the GVF snake is that it can capture a snake from a long range from both sides of the object boundaries and can force it into concavities. In order to calculate the GVF field, we need first to calculate the edge map *f*(*x*, *y*) given as

(10) $$\begin{array}{c} f\left(x,y\right)=-E_{\text{ext}}^{i}\left(x,y\right), \end{array}$$

where *i* = 1,2, 3, and  4. The field ∇*f* has vectors pointing towards the edges but these vectors have a very narrow range, and in the image where intensity is constant ∇*f* is zero, and no information is available about the nearby or distant edges in that region. The GVF field provides information about regions where *I*(*x*, *y*) is constant by the method of computational diffusion, defined as a vector field *V*(*x*, *y*) = (*u*(*x*, *y*), *v*(*x*, *y*)) which minimizes the energy function given below:

(11) $$\begin{array}{c} \varepsilon =\iint \mu \left(u_{x}^{2}+u_{y}^{2}+v_{x}^{2}+v_{y}^{2}\right)+{\left|\nabla f\right|}^{2}{\left|V-\nabla f\right|}^{2}dx\;dy. \end{array}$$

This formulation works on the standard method of making the data smooth. We can see that when |∇*f*| is small, then the energy is dominated by the partial derivatives of the vector field giving a smooth field (this term is also called the smoothing term) and when |∇*f*| is large the second term (known as the data term) is dominant which is minimized by setting *V* = ∇*f*. The parameter *μ* gives the trade-off between these two terms and must be set according to the amount of noise present in the image. The more is the noise the larger is the *μ*. 

The solution of (11) can be obtained from the calculus of variations by solving the following pair of Euler equations [8]:

(12) $$\begin{array}{c} \mu \nabla u^{2}-\left(u-fx\right)\left(fx^{2}+fy^{2}\right)=0,\\ \mu \nabla v^{2}-\left(v-fy\right)\left(fx^{2}+fy^{2}\right)=0. \end{array}$$

From the above two equations, it is clear that in a homogeneous region the second term of both equations becomes zero as the gradient is zero and here *u* and *v* both are determined by the first term which gives rise to generation of information from the data taken from boundaries. These two equations can be solved by treating *u* and *v* as a function of time, and solution of these equations is given below:

(13) ut(x,y,t)=μ∇2u(x,y,t)−(u(x,y,t)−fx(x,y))·(fx(x,y)2+fy(x,y)2),vt(x,y,t)=μ∇2v(x,y,t)−(v(x,y,t)−fy(x,y))·(fx(x,y)2+fy(x,y)2).

The solution of these two parabolic differential equations known as generalized diffusion equations is the desired solution of the Euler equations. For simplicity, we can rewrite the above pair of equations as follows:

(14) $$\begin{array}{c} u_{t}\left(x,y,t\right)=\mu \nabla^{2}u\left(x,y,t\right)-b\left(x,y\right)u\left(x,y,t\right)+c^{1}\left(x,y\right),\\ v_{t}\left(x,y,t\right)=\mu \nabla^{2}v\left(x,y,t\right)-b\left(x,y\right)v\left(x,y,t\right)+c^{2}\left(x,y\right), \end{array}$$

where

(15) $$\begin{array}{c} b\left(x,y\right)=f_{x}{\left(x,y\right)}^{2}+f_{y}{\left(x,y\right)}^{2},\\ c^{1}\left(x,y\right)=b\left(x,y\right)f_{x}\left(x,y\right),\\ c^{2}\left(x,y\right)=b\left(x,y\right)f_{y}\left(x,y\right). \end{array}$$

Since we know *f*_*x*_ and *f*_*y*_ from (10), therefore the values of *b*(*x*, *y*), *c* ^1^(*x*, *y*), and *c* ^2^(*x*, *y*) can be computed and fixed for the iterative process decided to find the values of *u* and *v*. In order to start the iterative process, let *i*, *j*, and *n* correspond to *x*, *y*, and *t*, respectively. Let *∇x* and *∇y* be the spacing between the pixels along *x* and *y* direction and *∇t* the time step for each iteration; then, the partial derivatives can be approximated as given below:

(16) $$\begin{array}{c} u_{t}=\frac{1}{\nabla t}\left(u_{i}^{n+1}-u_{i,j}^{n}\right),\\ v_{t}=\frac{1}{\nabla t}\left(v_{i}^{n+1}-v_{i,j}^{n}\right),\\ \nabla^{2}u=\frac{1}{\nabla x\nabla y}\left(u_{i+1,j}^{}+u_{i,j+1}^{}+u_{i-1,j}+u_{i,j-1}-4u_{i,j}\right),\\ \nabla^{2}v=\frac{1}{\nabla x\nabla y}\left(v_{i+1,j}^{}+v_{i,j+1}^{}+v_{i-1,j}+v_{i,j-1}-4v_{i,j}\right). \end{array}$$

By putting values from (15) to (16) into (14), we get the iterative solution to the GVF as follows [17]:

(17) ui,jn+1=(1−bi,j∇t)ui,jn+r(ui+1,jn+ui,j+1n   +ui−1,jn+ui,j−1n−4ui,jn)+ci,j1∇t,vi,jn+1=(1−bi,j∇t)vi,jn+r(vi+1,jn+vi,j+1n   +vi−1,jn+vi,j−1n−4vi,jn)+ci,j2∇t,

where *r* = *μ*∇*t*/(∇*x*∇*y*).

The solution of (17) is stable when a restriction on the stepsize of *r* ≤ (1/4) is maintained. The restriction on the stepsize can also be written as ∇*t* ≤ ∇*x*∇*y*/(4*μ*). After calculating the value of the GVF field, we can put the value of *V*, which is a function of *u* and *v*, back into (9). Now by solving (10) by the implicit finite difference method, a solution in the form of GVF snake can be obtained.

## 3. Snakes, Coordinate Regeneration, and Application to Complex Object

Ordinary snake has a serious problem of short range, and this is a big hurdle in its convergence. For improved convergence using ordinary snakes, one needs to initialise the snake very close to the original boundary of the object. GVF presents a solution with extended field, and the snake can be initialised away from the object boundary. It will be shown later in this paper that for object with complex concavities, GVF would fail to converge to original object boundary even in the presence of extended field, and the ultimate solution would come from the coordinate regeneration method (CRM). In CRM, field is first extended by convolving the image with a Gaussian function of large standard deviation. That will extend the field range, but at the same time it will blur the edges making it hard for the snake to converge properly to the original object concavities. Ordinary snake method is applied to the extended field, and a rough boundary is determined. This is a first step in CRM. To refine the boundary, the coordinates obtained from the rough boundary are interpolated to generate coordinates in the gap between two consecutive data points. This gives the rough boundary obtained in the first step more data points. This rough boundary is then used as initial snake in the second step of CRM but with the less extended field obtained with a Gaussian function of smaller size. Ordinary snake is applied, and a refined boundary is obtained. In the third step of CRM, coordinates are generated in the gaps between the data points of the refined boundary and presented as initial snake to an even less extended field, and an ordinary snake method is applied. This process is repeated unless convergence is achieved. A more complex boundary would require more stages of CRM. Here, we present an example with object consisting of complex concavities to demonstrate the concept of CRM. 

**Figure 1:**
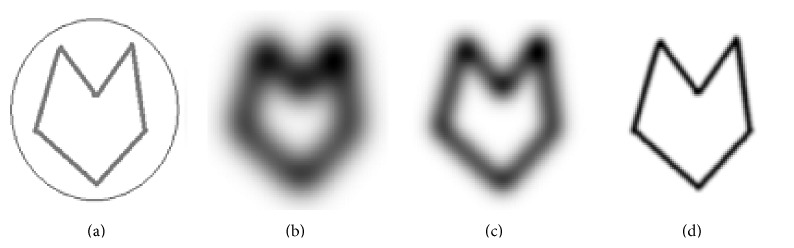
(a) An image with complex concavities; ((b)–(d)); display of image in (a) after Gaussian blur with standard $\sigma =\left(n-1\right)/6\sqrt{2}$ for *n* = 16, 10, and 2, respectively.

An object with complex concavities with its Gaussian blur images is shown in Figure 1.  Figure 1(a) shows an image with complex concavities, and the outer circle shows the boundary of the initial snake. Figures 1(b), 1(c), and 1(d) show the Gaussian blur images of the original object in Figure 1(a) obtained by the convolution of the object in Figure 1(a) with a Gaussian blur function with a standard deviation $\sigma =\left(n-1\right)/6\sqrt{2}$ for *n* = 16, 10, and 2, respectively. The blurring depends on the size of the kernel which is controlled by *n*. The edges in Figure 1(b) are more blur as compared with edges in Figure 1(c). The same is true for the image in Figure 1(c) in comparison with the image in Figure 1(d). It is clear from the images shown in Figure 1 that when the length of kernel is large (*n* = 16), more blurring is produced and concavities become less sharp, while concavities are much better seen in Figure 1(d) where the length of kernel is small. 

The size of the first or the largest Gaussian kernel depends on how far away the snake is initialised. If a snake has to be initialised say *m* pixels away from the farthest boundary in a given object, then the size of the largest Gaussian kernel should be such that the blurring effect on the farthest boundary should exceed well the *m* pixels range which means that the field due to the farthest boundary should go beyond the *m* pixels range around it. A snake initialised anywhere in the range of *m* pixels would be attracted to the diffused object boundaries, (see Figure 3). Further refinement in the object boundaries would require the use of a smaller Gaussian kernel which means a smaller field around the object boundaries or attraction of the snake to more accurate boundaries. Subsequent use of smaller and smaller Gaussian kernels would finally lead to the original object boundaries, and at the end, a Gaussian blurring kernel of size 2 pixels by 2 pixels would converge the snake to the original object boundary. A Gaussian blurring kernel of size *M* by *M* pixels would lead to a blurring effect extended by *M* pixels along the horizontal and *M* pixels along the vertical directions in an image about a point on a boundary line. 

**Figure 2:**
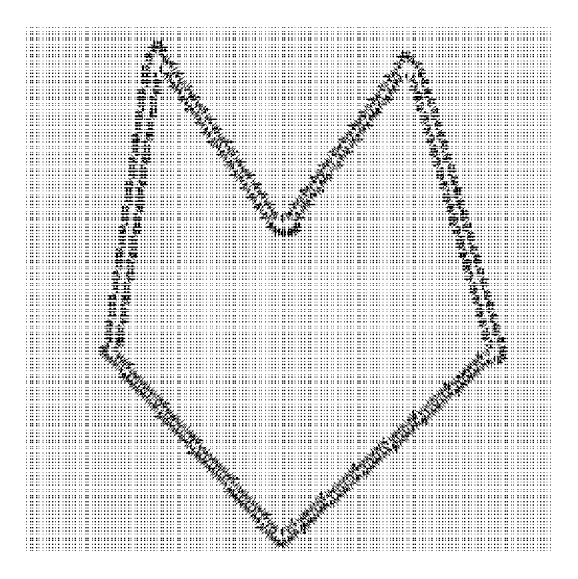
Display of vector field of image shown in Figure 1(a).

**Figure 3:**
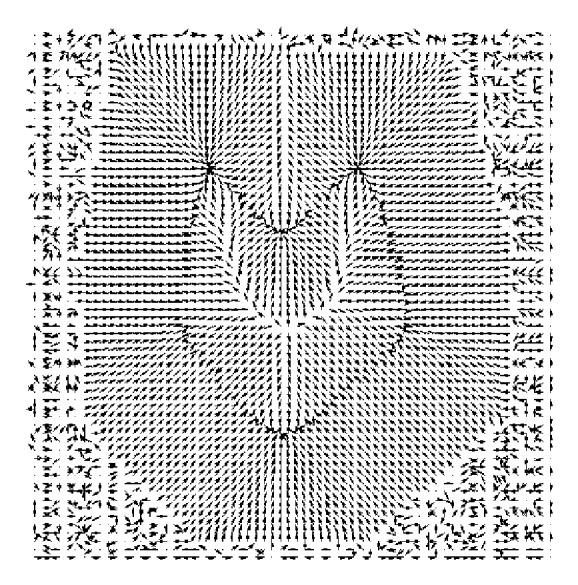
Display of vector field of image shown in Figure 1(c).

Figure 2 shows the vector field of the image shown in Figure 1(a). This image has dimension of 256 by 256 pixels and from the display it is clear that it has a very short vector field range, so it is impossible for the field to attract the initial snake if the snake is initiated away from the original boundary. The reason why the ordinary snakes fail to converge to original boundary is obviously the short range field shown in Figure 2. Figure 3 shows the vector field of image shown in Figure 1(c). The field in Figure 3 is well extended in comparison with the field shown in Figure 2. The field in Figure 2 is short range, and convergence can only be achieved if the initial snake is lying within the field range. The direction of the field in Figure 3 is such that it can attract the snake initialised anywhere within the field. The boundary coordinates obtained using such a field will be quite different from the actual boundary shown in Figure 1(c) or in Figure 2. The Gaussian blur enhances the field, but at the same time it modifies the original object boundary. The boundary shown by the field in Figure 3 may be regarded as a rough boundary. 

**Figure 4:**
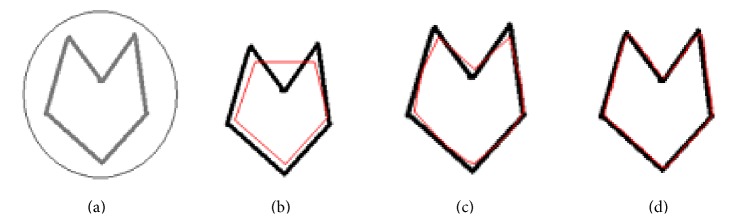
(a) Original object with initial snake encircling it, (b) snake on original object after first application of CRM (Gaussian blur with *n* = 16, 100 iterations), (c) snake on original object after second application of CRM (Gaussian blur with *n* = 10, 100 iterations), and (d) snake on original object after third application of CRM (Gaussian blur with *n* = 2, 100 iterations).

In CRM, blur versions of the original input object are prepared using Gaussian blur function. The snake is initialised not on the original object but on the most blur version of the input object (Figure 1(b)), and a rough boundary in the first step of CRM is determined as shown in Figure 4(b). The rough boundary obtained in the first step of CRM might be deficient in coordinates as the points constituting the original boundary might have shifted to the corners or have gathered at different places leaving behind gaps between the consecutive data points. This happens under the influence of external and internal forces. The gap between the consecutive data points in the rough boundary may be uneven: larger at one place or smaller at another place. Such a boundary cannot be fed to the second stage of CRM as an initial snake. The gap between the data points is filled using standard interpolation functions. The filling-up of gaps is regarded as coordinate regeneration. The rough boundary after implementation of coordinate regeneration is presented as initial snake to a relatively less blur image of the original object (image in Figure 1(c)). The new snake moves under the field shown in Figure 3 and converges to an improved boundary as shown in Figure 4(c). The boundary shown in Figure 4(c) is slightly deviated from the actual object boundary. This boundary after implementation of coordinate regeneration is presented as initial snake to a version of the object (image in Figure 1(d)) which is slightly blurred. The field corresponding to the image in Figure 1(d) is slightly extended version of the field shown in Figure 2. The field moves the snake to the required boundary as shown in Figure 4(d). 

**Figure 5:**
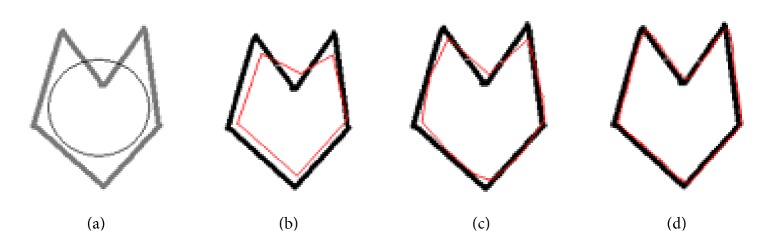
(a) Original object with initial snake partly inside and partly outside it, (b) snake on original object after first application of CRM (Gaussian blur with *n* = 16, 100 iterations), (c) snake on original object after second application of CRM (Gaussian blur with *n* = 10, 100 iterations), and (d) snake on original object after third application of CRM (Gaussian blur with *n* = 2, 100 iterations).

The main logic behind this modified concept of CRM is to use a bigger kernel *n* = 16 for the image, so that a broad range field can be obtained. Then, the ordinary snake method is applied which is capable of bringing the initial snake close to the boundary. In the second step a smaller kernel of length *n* = 10 is used to readjust the position of the snake achieved after the first step. The readjustment is achieved by introducing new coordinates on the curve obtained from the final result of the first stage. After this step, enough points are available for the forces to act and bring the snake into the concavities. In the third step, a very small kernel of length *n* = 2 is used and processed in a similar way as in the second step. Since the field range in the final stage is small, it will force the initial snake brought by the previous steps close to the original object to converge precisely to the actual object boundary. The snake finally converges to the complex concavities in the original object (Figure 4). In this example, the snake was initialised outside the object boundary. Figure 5 shows a set of results in which the snake is initialised partly inside and partly outside the image. The rest of the steps are the same as described earlier for the images in Figure 4. These two examples establish that CRM is capable of moving the snake around any kind of complex concavities.

## 4. Comparison of GVF Snake with Simple Snake Combined with CRM

GVF is a method for the extension of field in an image at the points where the actual field does not exist. It is a computational diffusion process in which the strong field at the edges is diffused by a method in which the sharpness of concavities is maintained. While working on this method, it has been observed that there is a limit for diffusion of data beyond which further diffusion is not possible. The main reason is that the data at these points have already attained a very small value that the further extension of these small values is not possible. It has also been observed that the extension of field by GVF, method is nonsymmetric. To get symmetric distribution of GVF it is needed to run the image in two steps. First pass will diffuse the data from right to left, and the second pass will diffuse the data from left to right making the diffusion of field symmetric. 

**Figure 6:**
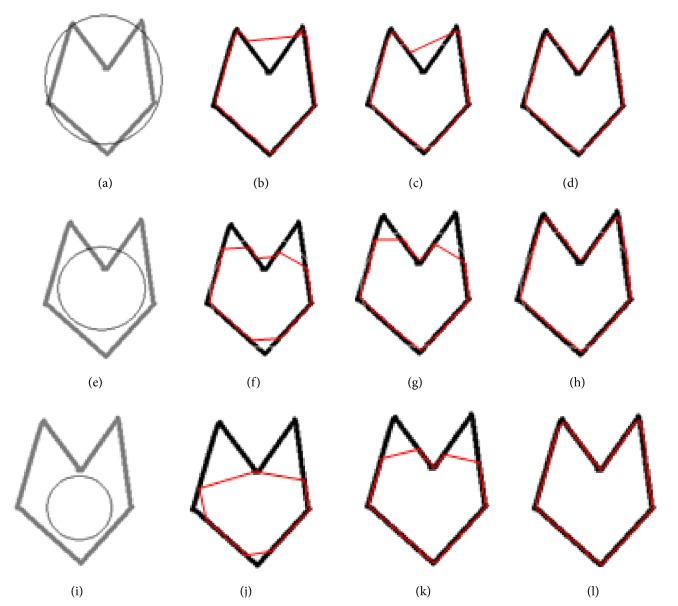
Comparison of GVF snake with ordinary snake coupled with CRM: ((a), (e), and (i)) input objects with initial snakes in black circles, ((b), (f), and (j)) convergence of final snake in 100 iterations using GVF method, ((c), (g), and (k)) convergence of snakes in 100 iterations during the first step of CRM and ((d), (h), and (j)) convergence of snakes in 100 iterations during the second step of CRM.

An important fact about the calculation of vector field either using simple snake method or using GVF is that the vector field is calculated using central derivative of the image along *x*-axis and *y*-axis. This derivative gives the perpendicular field directions at the image boundaries, but if we use forward or backward derivatives, then the forces appear in such a way that the directions of field along some orientations do not stay perpendicular, rather it becomes curved. The results of GVF snake which we are presenting in Figure 6 use symmetric GVF field based on central derivative. 

In Figure 6, we are comparing the GVF snake with ordinary snake coupled with CRM. The first column on the left in Figures 6(a), 6(e), and 6(i) shows input object with initial snake as black circles. The second column (Figures 6(b), 6(f), and 6(j)) shows the results obtained with GVF snake. The third column (Figures 6(c), 6(g), and 6(k)) shows results obtained in the first application of ordinary snake when coupled with CRM, and the forth column (Figures 6(d), 6(h), and 6(l)) shows results corresponding to the second application of CRM. Convergence was achieved well before 100 iterations in each case. Comparison reveals that the CRM is superior to GVF method. GVF method which is a well-reputed method could not give accurate boundary when tested on objects with complex concavities, whereas the proposed CRM method yields accurate results. In the presence of even more complex object, CRM is found to give ultimate solution to determine the boundary of objects of interests. 

## 5. Ordinary Snake with CRM for Optic Disc Boundary Detection

This section describes the application of active contours (snakes) in finding the boundary of the optic disc in a retinal image. The retinal images were coloured and were converted to red, green, and blue channel images. Red channel images were used in finding the vector field. The vector field pushes the initial boundary coordinates (initial snake) towards its final boundary coordinates (final snake). Before the application of active contours to retinal images, a retinal image or its optic disc area needs to be processed in order to clear the blood vessels from and around the optic disc. 

Blood vessels act as a distracter and modify the vector field in such a way that the final snake is deviated from the actual boundary of the optic disc. The grey channel and the green channel images have blood vessels with a higher contrast as compared with their red channel counterparts. Processing of grey and green channel retinal images for the removal of blood vessels from and around the optic disc requires varying image processing techniques from image to image, and the automation for active contours in determining the optic disc boundary may not be possible to achieve. We have chosen the red channel retinal images and have achieved automation on all the available images. 

**Figure 7:**
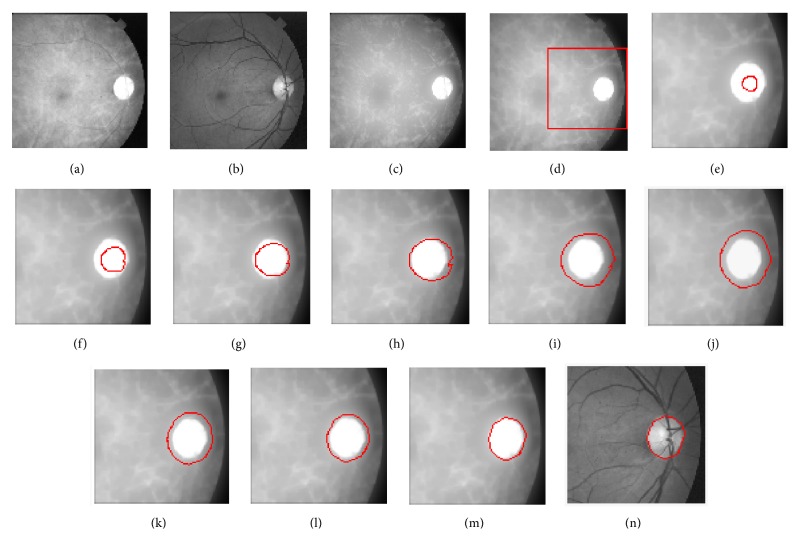
Boundary-fit to the red channel retinal image: (a) red channel retinal image, (b) green channel counterpart of image in (a), (c) maximum of the image in (a) and its Gaussian blurred image with *σ* = 24, (d) greyscale-close of the image in (c) with kernel of (10, 10) with all elements unity, (e) part of image in (d) indicated by red boundary of size (300, 300) with initial snake in red on optic disc, ((f), (g), (h), (i), (j), (k), and (l)) active contours after 15, 25, 35, 45, 55, 65, and 75 iterations, respectively, (m) convergence of final snake at 80th iteration on red channel, and (n) boundary of image in (m) shown on green channel image.

Red channel images also require some image processing for the removal of blood vessels. Typical red and green channel images are shown in Figures 7(a) and 7(b), respectively. The image in Figure 7(a) has an optic disc with a lower contrast of blood vessels. The area around the optic disc has variation in intensities and can cause the vector field to be directed randomly at some points resulting in the convergence of the final snake at points other than the optic disc boundary points. The ideal situation for the correct boundary convergence of the final snake requires two things: (a) the area of the optic disc and around it must be free of blood vessels and (b) the area around the optic disc must be smooth giving the optic disc sharp boundaries. The latter condition is especially important when the initial snake is lying outside the optic disc boundary. 

The edge enhancement of the optic disc in a red channel image and the smoothness of the area around the optic disc are achieved before initialisation of the snake. The image in Figure 7(c) has the optic disc boundary enhanced, and the area around the optic disc is smoothed as compared with the image in Figure 7(a). The image in Figure 7(c) is the maximum of the image shown in Figure 7(a) and the image obtained from Figure 7(a) by convolving it with the Gaussian kernel of size *σ* = 24. To remove the blood vessels from the optic disc in Figure 7(c), the greyscale-close of the image shown in Figure 7(c) was computed with a structuring element having dimensions 10, 10 with all the elements equal to unity. The resultant blood-vessel-free optic disc is shown in Figure 7(d). A patch of dimensions 300, 300 pixels taken from the image in Figure 7(d) is shown in Figure 7(e) with the initial snake shown in red on the optic disc. The images in Figures 7(f), 7(g), 7(h), 7(i), 7(j), 7(k), and 7(l) were taken at 15, 25, 35, 45, 55, 65, and 75 iterations, respectively. 

The final snake was converged to the optic disc boundary at the 80th iteration and is shown in Figure 7(m) on the red channel image and in Figure 7(n) on the green channel image. The image in Figure 7(e) is first convolved with a LOG filter of appropriate kernel size, and *f*_*x*_ (*x* derivative) and *f*_*y*_ (*y* derivative) are calculated from the resultant image. The field is oppositely directed at an edge boundary and forms a final convergence boundary for an initial snake initialised within or outside the boundary. 

In some cases, the red channel images even after preimage processing are left with some traces of blood vessels and the vector field is distorted within the optic disc, which may cause the final snake to converge to points other than the disc boundary points. The distortion in the vector field due to vessel traces leftover after pre-image processing within an optic disc can be avoided if a stronger field is set up within the optic disc. The higher the size of the kernel of the LOG filter the stronger is the vector field. A stronger vector field normally broadens the boundary of the optic disc around its centre, which will make the initial snake converge to the new boundary setup by the vector field. This situation is presented in Figures 7(h), 7(i), and 7(j) in which the snake is directed away from the actual boundary of the optic disc. 

The image in Figure 7(j) shows the situation in which the snake boundary is converged under a stronger vector field and where the forces towards and away from the boundary of the optic disc are balanced. At this stage, the vector field is changed by using a lower value of kernel size of the LOG filter. The lower value of kernel size brings the vector boundary closer towards the actual optic disc boundary, and the snake is now balanced to a boundary closer to the actual optic disc boundary and this situation is shown in Figure 7(k). Further lowering the value of the kernel size of the LOG filter brings the snake to the situation shown in Figure 7(l), and ultimately the final snake is converged to the exact boundary of the optic disc as shown in Figure 7(m). To automate the snake convergence on a variety of optic discs having different diameters, we used the kernel sizes for the LOG filter as {281,211,151,111,81,51,31,11} with number of iterations equal to 10 for each case. The other parameters used for the snake were Δ*s* = 1 (space iteration), Δ*t* = 0.01 (time iteration), *α* = 0.2, and *β* = 0. The technique described above to find the boundary around an optic disc using application of active contours (snakes) on a red channel retinal image is novel and fully automatic and gives good results, when tested on large number of retinal images. A point on an optic disc is required to initialise a snake, and this point can be determined by locating a point with a maximum variance.

In the first step of CRM, a larger Gaussian blurring kernel is used which shifts the virtual boundary outside the original boundary, and the initial snake expands first. In the subsequent stages where the size of the kernel is reduced, the virtual boundary tends to move towards the original boundary, and when the blurring kernel size is very small, the virtual boundary overlaps with the original boundary and so the final snake converges or shrinks to the original boundary. The images from (e) to (i) in Figure 7 correspond to the first step of CRM where the virtual boundary lies outside the original boundary. In the rest of the images, the kernel size is reduced, and so the snake shrinks. 

**Figure 8:**
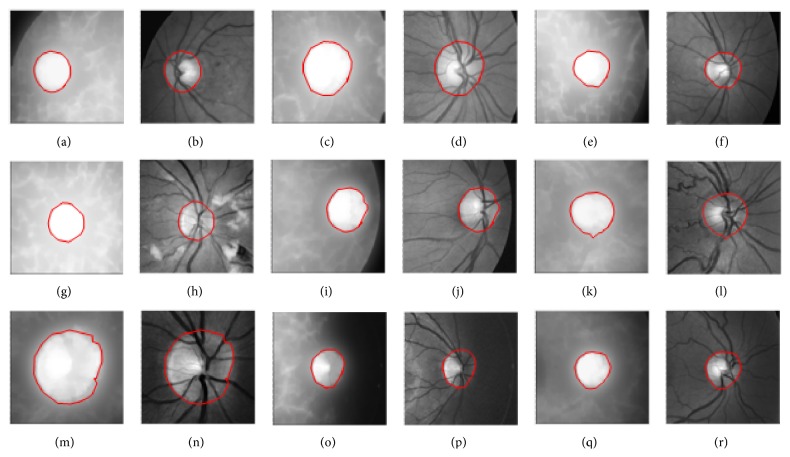
Boundary-fit to optic discs using active contours (snakes) with CRM, images in (a), (c), (e), (g), (i), (k), (m), (o), and (q) are red channel images with their green channel counterparts next to them.

Figure 8 presents red and green channel images fitted with final snakes similar to the images shown in Figures 7(m) and 7(n). The boundaries were found to fit quite reasonably in each case. We have tested the CRM on a variety of 70 different coloured retinal images and found the results satisfactory. Some of the results have been reported in Figure 8. To quantify the results, three human graders from the field of ophthalmology were chosen to draw boundaries around the optic discs in 70 retinal images, and a plenty of time was given for this job. Their results were processed, and boundary lines passing through the mid of their drawn line widths were selected for comparison with the boundary coordinates obtained with CRM method. The size of the optic disc was found to vary from 70 pixels to 126 pixels. The results of the three human graders were averaged and then rounded. The comparison revealed a maximum error of 2 pixels across the dimensions of optic discs. The error varied from 1.59% to 2.86% corresponding to optic disc of linear dimensions of 126 and 70 pixels, respectively. 

**Figure 9:**
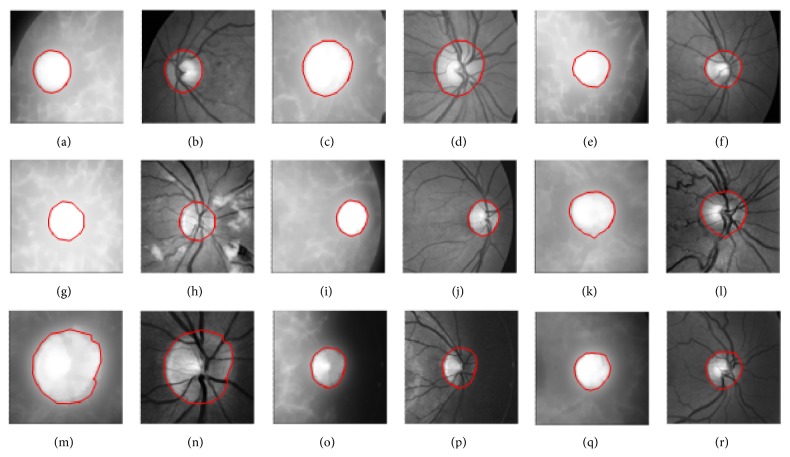
Boundary-fit to optic discs using GVF snake, images in (a), (c), (e), (g), (i), (k), (m), (o), and (q) are red channel images with their green channel counterparts next to them.

For comparison purposes, we have presented to GVF snake the images ((a), (c), (e), (g), (i), (k), (m), (o), and (q)) shown in Figure 8, and the results are given in Figure 9 on red and green channel images. The GVF snake parameters used in the results shown in Figures 6 and 9 were ∇*t* = 0.01; ∇*x* = ∇*y* = 0.5; *μ* = 1.5. The difference in the active contours in the corresponding images on the two sets of images shown in Figures 8 and 9 is very small and hard to be noticed with naked eye. It was found that GVF snake gives results very close to the results obtained with CRM. The mean % error in the diameters along different directions in the corresponding set of images was found to vary from 0 to 3. We may therefore conclude that GVF gives reasonably accurate results when tested on retinal images but at the expense of larger computational time in comparison with CRM. However, CRM is superior to GVF when tested on objects with complex concavities. 

## 6. Conclusion

This paper has described a method for locating the boundary of an optic disc. Simple snake (active contours), GVF snake and simple snake based on coordinate regeneration method (CRM) were presented, and a comparison between the GVF snake and CRM was given. In each case, a point on the optic disc is required to initial a snake. A point on an optic disc can be determined by computing the maximum value of variance in a retinal image. This works only in a normal retinal image where the optic disc is assumed to have the brightest intensity in a retinal image giving the highest contrast for vessels. Calculation of variance image can be adopted for abnormal images and in the presence of exudates bigger and brighter than the optic disc to locate a point on it. A point on the optic disc can also be located by blood vessel tracking using its binary tree image. Optic disc is the origin of all the blood vessels in a retinal image, and the possibility that a point is located on the optic disc by the method of blood vessel tracking is relatively higher. After a point is located on optic disc in a retinal image, a snake can be initiated in the form of a circle around that point. 

The initial snake under the influence of forces (vector field) drifts towards the actual boundary of the optic disc where the forces within the optic disc are balanced by the forces outside the optic disc. We have implemented three schemes for the active contours, namely, ordinary snakes, gradient vector flow (GVF) snakes, and snakes based on CRM. Merits and demerits of each technique have been discussed and illustrated with examples of objects with complex concavities. We have introduced a novel concept of coordinate regeneration that when combined with ordinary-snake eliminates the need for GVF snakes. We have established that our method of snakes with coordinate regeneration gives better results and ultimately converges to the desired boundaries where the GVF snakes fail. We have extended the ordinary snake method to successfully locate the boundary of complex objects and the boundary of optic discs in retinal images. The preimage processing of optic disc and the region around it has been given in a simple and more effective way as compared with methods suggested in the literature [2]. Our method to locate optic disc boundary using snakes is fully automatic and comprehensive. 

The GVF snake is a computationally expensive technique in comparison with our CRM based technique. The time taken by GVF for the processing of 70 retinal images in automatic mode using Pentium(R) D CPU 3.4 GHz, 2 GB of RAM, Microsoft Windows XP Professional Service Pack 2 Version 2002 and Mathematica 4.1 in a stand-alone mode was found to be 41 minutes, 21 seconds, and 99 milliseconds. With the application of CRM on the same set of images included the preprocessing time was 19 minutes, 12 seconds, and 60 milliseconds. The performance of GVF snake using different preprocessing criteria [2] was lower than the performance given by CRM using our novel preprocessing techniques. However, if GVF is implemented with our proposed preprocessing methods, then the performance of GVF approaches to that of CRM at the expense of more computational time (53 minutes, 10 seconds, 101 milliseconds). We have demonstrated that GVF does not give accurate results when tested on objects with complex concavities and the performance of CRM methods remains superior. 

Blood vessels are the main distracters of snake when active contour methods are applied to optic disc boundary localization. This problem has been addressed successfully by our preprocessing methods which are applied prior to the application of active contours on retinal image. We believe that our CRM snake can successfully be implemented in locating boundaries of different object species in medical images and in other domains of image modalities. We also believe that the CRM snake will prove to be a promising technique.
